# Promotion of nutritional behaviors in the prevention of cardiovascular diseases: application of the health belief model in primary health care centers

**DOI:** 10.1186/s12875-023-02248-6

**Published:** 2023-12-18

**Authors:** Nasrin Midjani, Fatemeh Alsadat Hossaini, Nader Sharifi

**Affiliations:** 1 Research Center for Social Determinants of Health, Jahrom University of Medical Sciences, Jahrom, Iran; 2 Department of Public Health, Khomein University of Medical Sciences, Khomein, Iran

**Keywords:** Cardiovascular diseases, Primary health care, Education, Diet, Health belief model

## Abstract

**Background:**

Cardiovascular diseases (CVD) are the most important cause of death in many countries of the world, including Iran. This study aimed to investigate the effect of educational intervention based on the Health Belief Model (HBM) on the promotion of nutritional behaviors to prevent CVD among the all people (aged 30–59 years).

**Methods:**

This semi-experimental study was conducted on all people (aged 30–59 years) referring to the primary healthcare centers of Jahorm city, southern Iran, from September 2021 to July 2022. In this study, 100 participants (50 participants for the intervention group and 50 for the control group) were selected using a multistage cluster random sampling method. The data collection tool was a questionnaire with confirmed validity and reliability. Also, the food consumption frequency checklist was used. The educational intervention included holding four sessions, each lasting for 60 min. The methods used for training included lectures, questions and answers, and group discussions. Before the intervention and three months after, the intervention and control groups completed the questionnaire. The obtained data were analyzed by SPSS 17.

**Results:**

after the educational intervention, the mean score of knowledge (4.84 ± 0.84; 2.76 ± 1.41; p < 0.001), perceived susceptibility (9.52 ± 1.81; 8.76 ± 1.9; p = 0.004), perceived severity (14.78 ± 1.66; 13.80 ± 2.23; p = 0.015), perceived benefits (10.66 ± 1.79; 7.52 ± 1.99; p < 0.001), perceived barriers (5.92 ± 2.81; 12.68 ± 10.24; p < 0.001) and practice (178.78 ± 14.35; 147.36 ± 13.19; p < 0.001) showed a significant difference between the intervention and control groups.

**Conclusion:**

The results showed that the educational intervention effectively improved people (aged 30–59 years) knowledge and HBM constructs to prevent CVD. Also, improving people’s performance regarding CVD prevention behaviors will be successful by implementing an educational intervention based on the HBM.

**Supplementary Information:**

The online version contains supplementary material available at 10.1186/s12875-023-02248-6.

## Introduction

This study aims to investigate the impact of an educational intervention based on the Health Belief Model on promoting nutritional behaviors to prevent Cardiovascular diseases (CVD) among all people (aged 30–59 years). CVD is the most important cause of death and one of the leading causes of disability in many countries of the world, including Iran [1, 2]. It is predicted that CVD deaths will reach 23.6 million people worldwide by the end of 2030, and it will account for 44.8% of deaths in Iran [3]. In recent years, the age of CVD in Iran has decreased [4]. Some changeable factors (diet, physical activity, smoking, and alcohol consumption) related to people’s lifestyle are involved in the incidence of CVD, one of the most important of which is diet [5–7]. Modifying lifestyle is the most important method of preventing CVD, which can reduce many risk factors related to these diseases [8]. Modifying food patterns plays a vital role in preventing cardiovascular diseases, especially heart infarction; the Mediterranean diet is associated with reducing the risk of CVD [9, 10]. Global analyses have attributed millions of deaths from cardiovascular disease to various dietary factors, the most common being low consumption of fruits, whole grains, vegetables, nuts, and seeds and high salt intake [11]. This is while Western diet patterns increase mortality due to cardiovascular diseases [12]. Education is very important in modifying nutritional behaviors to prevent health problems [13]. The most effective educational programs are based on theory-based approaches rooted in behavior change models [14]. The Health Belief Model (HBM) is one of the most widely used models of health behavior change, often used to prevent diseases [15]. The HBM includes perceived susceptibility (a person’s abstract beliefs regarding the possibility of contracting an illness), perceived severity (a person’s abstract belief about the severity of injury that can occur to him as a result of acquiring a disease), perceived benefits (confidence in the advantages of the proposed methods to reduce the risk or worsening of the disease), perceived barriers (beliefs about the natural and perceived costs of pursuing a new behavior), cues to action (accelerating forces that make a person feel the need to act) and self-efficacy (a person’s confidence in his abilities to perform and follow a behavior successfully) [16, 17].

## Method

### Study design

This semi-experimental study was conducted on all people (aged 30–59 years) referring to the primary health care centers of Jahorm city located in Fars province of southern Iran from September 2021 to July 2022. A relatively high level of obesity and cardiovascular problems have been recorded in people over 30 years old in Jahrom city [18]. Primary health care centers are the first level of community contact to receive preventive and therapeutic services in Iran. In these centers, accessing the community’s people and implementing health education programs is possible.

Assuming a target difference of 0.4, SD 0.5, and standard difference of 0.8, and taking into account the confidence interval of 95% and power of 80% [19], and assuming the equal number of samples in both groups using Altman Nomogram, the sample size in the intervention and control groups was calculated to be 50 people (100 people in total). Sampling was a multistage cluster random sampling in which primary health care centers of Jahrom city were considered as separate clusters, and two centers were randomly selected. Then, to prevent contact between the intervention group and the control group and the transfer of educational content between the two groups, one center for the intervention group and another for the control group were randomly selected. In the next stage, the study participants were randomly chosen among the all people (aged 30–59 years) referring to each center. Inclusion criteria include All men and women (30–59 years old) referring to primary health care centers in Jahrom city who did not have a confirmed history of CVD. Exclusion criteria include Failure to complete the written consent form to participate in the study, Failure to respond to the questionnaires fully, and absence of more than one session in the educational program in the intervention group.

### Instrument and data collection

The questionnaire used in this study was designed by Tavassoli et al. in 2013 in Iran, and its validity and reliability were measured [19]. This questionnaire was based on the HBM, and the first part included questions related to demographic characteristics (gender, age, education level, family size, marital status, occupation, average monthly household income, and smoking), the second part included six knowledge questions, four perceived susceptibility (person’s perception of the possibility of danger) questions, four perceived severity person’s (perception of the seriousness of the risk) questions, three perceived benefits (person’s perception of the effectiveness of measures to reduce the risk of the disease) questions, and seven perceived barriers (person’s perception of the obstacles to performing health measures) questions. In the knowledge questions, the correct answer was given a score of 1; the rest were zero; the highest score was 6, and the lowest score was zero. The questions of perceived susceptibility, perceived severity, perceived benefits, and perceived barriers were designed as a 5-point Likert scale (completely agree, agree, neither agree nor disagree, disagree, and completely disagree). The highest score for each perceived susceptibility and severity construct was 20, and the lowest was 4. The highest score for the perceived benefits construct was 15, the lowest score was 3, the highest score for the perceived barriers construct was 35, and the lowest score was 7. To check the reliability of the questionnaire in this study, 30 people (aged 30–59 years) completed the questionnaire, and its reliability was measured using Cronbach’s alpha scale. The values obtained for knowledge were 0.77, perceived sensitivity 0.8, perceived severity 0.82, perceived benefits question 0.91, perceived barriers 0.79, and the whole questionnaire was 0.82.

Also, to measure people’s practice in the field of diet, the food consumption frequency checklist was used, which was prepared in the form of consumption of each of the desired foods with daily, weekly, and never options. (Have you consumed the following foods in the past week? How many times if used? Milk, yogurt, cream, jam/honey, cheese, bread, baguette, rice, all kinds of kebabs, pasta, chicken, fish, canned fish, lentils/beans, sausages, eggs, stews, soups, broth, potatoes, pizza, pickles, tomatoes, citrus fruits, other fruits, puffs, sweets, Nuts, ice cream, chocolate, tea, coffee, soda, dates, tamarind).

To complete the questionnaires, the people of the intervention and control groups were invited to the primary health care centers, and they completed the questionnaires under the supervision of the interviewers. At first, the study’s objectives were explained to the participants, who completed a written consent form. Then, the questionnaires were conducted by the people of the intervention and control groups. Then, the obtained information was analyzed, and educational materials and content were prepared based on educational objectives, using reliable sources and with the advice of cardiologists. Educational content regarding nutritional behaviors to prevent CVD was designed as HBM constructs. Then, the educational intervention was done for the intervention group, while no intervention was done for the control group.

### Educational intervention

Educational content was designed to improve nutritional behaviors to prevent CVD based on HBM constructs. The content included the following: low-fat diets, including low-fat meats low-fat dairy products, and emphasizing daily consumption of vegetables and fruits. A low-carb diet contains recommendations for a Mediterranean diet, including high consumption of omega-3 fatty acids from fish and plant sources, consumption of fruits, fresh seasonal vegetables, whole meal bread and cereals, nuts, olive oil, and legumes; Providing body protein from fish, chicken and eggs instead of red meat. Avoid harmful substances, including salt, sweets, carbonated drinks, and alcohol [20–22]. The educational content was reviewed and approved by a nutritionist and a cardiologist.

The educational intervention included holding four sessions, each lasting for 60 min. The methods used for training included lectures, questions and answers, and group discussions. Pamphlets and text messages were also used to repeat educational materials. Training sessions were held weekly. SMS was sent twice to each person in the interval between meetings. After the end of the training sessions, sending SMS twice a week continued for a month. At the end of the research, a training course was held to improve CVD prevention nutritional behaviors for the control group.

The people of the intervention and control groups were invited to the primary health care centers three months after the completion of the educational intervention, and the questionnaires were filled out again by them. It should be noted that this was the last stage of follow-up and completion of the questionnaire by the participants.

### Statistical analysis

The data was analyzed using SPSS 17. Kolmogorov–Smirnov test was used to check the normality of the data. Demographic variables were compared between the two groups using the chi-square test. Paired t-test and independent t-test were used to reach within and between groups.

## Results

The highest age frequency in the intervention group was 41–50 years old (46%); in the control group, it was 30–40 years old (44%). Most participants in the intervention (90%) and control (86%) groups were married. The highest education frequency in the intervention group (36%) and the control group (50%) was university level. The highest frequency of occupation of the people in the intervention group was housekeeper or unemployed (42%), and in the control group, self-employed (50%). The results showed no significant difference between the intervention and control groups regarding demographic variables, and the intervention and control groups were identical (Table 1).

**Table 1:** Comparison of frequency of demographic variables between intervention and control groups

Variables		Intervention group	Control group	p-value*
		N%	N%	
Gender	Male	18(36)	20(40)	0.321
	Female	32(64)	30(60)	
Age	30–40	22(44)	22(44)	0.207
	41–50	17(34)	23(46)	
	> 50	11(22)	5(10)	
Marital status	Single	5(10)	7(14)	0.538
	Married	45(90)	43(86)	
Education	elementary school	14(28)	12(24)	0.401
	middle school	7(14)	3(6)	
	diploma	11(22)	10(20)	
	university	18(36)	25(50)	
family size	2	11(22)	9(18)	0.362
	3	21(42)	15(30)	
	4	11(22)	14(28)	
	5	2(4)	7(14)	
	6	5(10)	5(10)	
Employment status	housekeeper or unemployed	18(36)	9(18)	0.102
	employed	17(34)	25(50)	
	self-employed	15(30)	16(32)	
Monthly household income	Under 5 million tomans	19(38)	12(24)	0.165
	5–10 million tomans	19(38)	18(36)	
	Above 10 million tomans	12(24)	20(40)	
Smoking	No	46(92)	38(76)	0.134
	Yes	4(8)	12(24)	

*chi-square test

The results showed that the difference in the mean score of knowledge, perceived susceptibility, perceived severity, perceived benefits, perceived barriers and practice between the intervention and control groups was not significant before the educational intervention. While after the educational intervention, the mean score of knowledge (4.84 ± 0.84; 2.76 ± 1.41; p < 0.001), perceived susceptibility (9.52 ± 1.81; 8.76 ± 1.9; p = 0.004), perceived severity (14.78 ± 1.66; 13.80 ± 2.23; p = 0.015), perceived benefits (10.66 ± 1.79; 7.52 ± 1.99; p < 0.001), perceived barriers (5.92 ± 2.81; 12.68 ± 10.24; p < 0.001) and practice (178.78 ± 14.35; 147.36 ± 13.19; p < 0.001) showed a significant difference between the intervention and control groups (Table 2).

**Table 2:** Comparison of knowledge, perceived susceptibility, severity, benefits, barriers, and practice in intervention and control groups

Variables		Intervention (Mean ± SD)	Control (Mean ± SD)	P*
Knowledge	Before intervention	1.94 ± 1.08	2.1 ± 1.18	0.48
	After intervention	4.84 ± 0.84	2.76 ± 1.41	< 0.001
Perceived susceptibility	Before intervention	8.32 ± 1.99	8.92 ± 2.21	0.157
	After intervention	9.52 ± 1.81	8.76 ± 1.91	0.004
Perceived severity	Before intervention	14.36 ± 1.99	13.86 ± 2.12	0.228
	After intervention	14.78 ± 1.66	13.80 ± 2.23	0.015
Perceived benefits	Before intervention	7.20 ± 1.81	7.30 ± 2.32	0.81
	After intervention	10.66 ± 1.79	7.52 ± 1.99	< 0.001
Perceived barriers	Before intervention	9.88 ± 2.76	10.96 ± 4.39	0.144
	After intervention	5.92 ± 2.81	12.68 ± 10.24	< 0.001
Practice	Before intervention	148.34 ± 11.77	144.12 ± 11.42	0.072
	After intervention	178.78 ± 14.35	147.36 ± 13.19	< 0.001

*Independent T-test

On the other hand, the results showed that in the intervention group, the mean score of knowledge (1.94 ± 1.08; 4.84 ± 0.84; p < 0.001), perceived susceptibility (8.32 ± 1.99; 9.52 ± 1.81; p = 0.003), perceived severity (14.36 ± 1.99; 14.78 ± 1.66; p = 0.043), perceived benefits (7.20 ± 1.81; 10.66 ± 1.79; p < 0.001), perceived barriers (9.88 ± 2.76; 5.92 ± 2.81; p < 0.001) and practice (148.34 ± 11.77; 178.78 ± 14.35; p < 0.001) after the intervention increased significantly compared to before the intervention; While in the control group, no significant difference was observed in any of these variables (Table 3).

**Table 3:** Comparison of knowledge, perceived susceptibility, severity, benefits, barriers, and practice before and after intervention

Variables		Before intervention (Mean ± SD)	After intervention (Mean ± SD)	P*
Knowledge	Intervention	1.94 ± 1.08	4.84 ± 0.84	< 0.001
	Control	2.1 ± 1.18	2.76 ± 1.41	0.11
Perceived susceptibility	Intervention	8.32 ± 1.99	9.52 ± 1.81	0.003
	Control	8.92 ± 2.21	8.76 ± 1.91	0.498
Perceived severity	Intervention	14.36 ± 1.99	14.78 ± 1.66	0.043
	Control	13.86 ± 2.12	13.80 ± 2.23	0.472
Perceived benefits	Intervention	7.20 ± 1.81	10.66 ± 1.79	< 0.001
	Control	7.30 ± 2.32	7.52 ± 1.99	0.411
Perceived barriers	Intervention	9.88 ± 2.76	5.92 ± 2.81	< 0.001
	Control	10.96 ± 4.39	12.68 ± 10.24	0.230
Practice	Intervention	148.34 ± 11.77	178.78 ± 14.35	< 0.001
	Control	144.12 ± 11.42	147.36 ± 13.19	0.100

*Paired t-test

## Discussion

The present study investigated the effect of an educational intervention based on the HBM on promoting nutritional behaviors that prevent CVD in the all people (aged 30–59 years) in Jahrom city. According to the results, there was no significant difference between the intervention and control groups in terms of demographic characteristics and HBM constructs before the educational intervention. Of course, because random allocation was not done in selecting the members of the groups, it is not possible to be sure that the characteristics of the two groups are identical, and there is a possibility of selection bias. The results showed that the average knowledge score in the intervention group increased significantly compared to the control group after the educational intervention. These results align with the results of Tavassoli [19] and Zainali [23] studies. Abedi’s study aimed to investigate the effect of lifestyle change on cardiac risk factors using the HBM. It showed that at the end of the study, all instruments of the HBM increased significantly in the intervention group compared to the control group. Still, the knowledge of the intervention group did not change significantly [24]. Improving people’s knowledge provides the basis for improving attitudes and adopting appropriate behaviors regarding risk factors and prevention of CVD. Therefore, improving people’s knowledge as a result of educational intervention seems very important and emphasizes the need to pay attention to training people in primary health care centers.

People’s access to information related to lifestyle modification requires the use of appropriate methods to educate people. The effectiveness of health education programs depends a lot on using a suitable theory in education [25]. It seems that HBM has been able to have a suitable effect in improving people’s knowledge in preventing CVD.

The results showed that after the educational intervention, there was a significant difference between the intervention and control groups in the mean score of knowledge, perceived susceptibility, perceived severity, perceived benefits, and practice regarding nutritional behaviors that prevent CVD. Also, the mean score of perceived barriers in the intervention group was significantly reduced compared to the control group.

Kheiri’s study to measure the effectiveness of the educational intervention based on the HBM on the prevention of CVD showed that the difference in the mean scores of perceived susceptibility, perceived severity, perceived benefits, and preventive behaviors significantly increased. Perceived barriers decreased in the intervention group, compared to the control group [26], which is consistent with the results of the present study. Also, Kahnooji’s study on the primary health care center staff aimed to effect educational intervention based on HBM to promote CVD preventive behaviors showed that the educational intervention improved the HBM constructs of the participants [27]. It should be said that the results of the present study and similar studies indicate that people’s perception of various aspects of CVD is at a low level, and educational intervention can improve different HBM constructs well. Another noteworthy point is that the perceived barriers score was significantly higher in the control group after the educational intervention. Many people in society seem to have problems and obstacles in adopting the correct health behavior in front of them, which can be reduced with proper education.

In the present study, the changes in the practice mean score of people regarding nutritional behaviors that prevent CVD in the intervention group have increased significantly more than the control group. In line with these results, in Shojaeizadeh’s study, students’ practice in preventing CVD improved significantly [28]; also, in the Shoja Fard study, nutritional behavior significantly improved in the intervention group [29]. In Shojaei’s study, the educational intervention based on HBM could not improve people’s practice in preventing cardiovascular problems [30]. Regarding these required results, it should be acknowledged that creating behavior change is a time-consuming and challenging process that requires a coherent program. Therefore, educational interventions should be designed and implemented considering the appropriate behavior change strategy and maintenance strategy.

According to proven psychological principles, human inclinations determine their behavior [31]. Therefore, when people’s education and attitude modification in society is not done enough, people choose behaviors based on pleasure seeking and basic desires, not on maintaining health. This problem is well evident in the control group. Because of the lack of proper educational planning in society and cultural poverty in this field, no improvement in knowledge, behavior, and HBM constructs has been observed in the control group.

One of the strengths of the current study was the good cooperation of the primary healthcare centers’ officials in the research implementation. Also, using questionnaires that covered all stages of behavior change from knowledge to practice is another strength of the study. One of the limitations of this study was the absence of two constructs of cues to action and self-efficacy in the questions of the standard questionnaire used. However, in the educational intervention, these two HBM constructs were also considered. Another limitation of the study was the low literacy of some participants, which required the completion of the questionnaires with the help of interviewers. The lack of long-term follow-up to investigate the creation and maintenance of desired knowledge and behavior is another limitation of the present study. The type of intervention in the current study was educational. In this type of study, there should not be a relationship between the compared groups Because it can cause the transfer of educational information and distort the result. Therefore, random allocation was not possible.

## Conclusion

This study showed that the educational intervention effectively improved the knowledge and HBM constructs in people (aged 30–59 years) to prevent CVD. The knowledge available in society cannot motivate people to adopt CVD-preventive behavior. Therefore, planning for social and individual interventions to increase sensitivity, intensity, and perceived benefits and reduce perceived barriers in this field will be helpful. Also, improving people’s performance regarding CVD prevention behaviors will be successful by implementing an educational intervention based on the HBM. Therefore, it is suggested to design and implement training programs based on this model in primary health care centers that cover most people in the community to reduce the risk factors and prevent CVD. It is recommended that in future studies, the adoption of preventive behaviors should be followed for a long time so that the effect of intervention based on the model on the creation and maintenance of the behavior is revealed.

### Electronic supplementary material

Below is the link to the electronic supplementary material.

**Supplementary Material 1:** An example of SMS sent

**Supplementary Material 2:** An example of text messages sent (translated from Persian)

## Data Availability

The datasets generated and analyzed during the current study are not publicly available because they contain raw data from study participants, and sharing these data requires participants’ permission. But are available from the corresponding author on reasonable request.
