# Supplementary material for: Promotion of nutritional behaviors in the prevention of cardiovascular diseases: application of the health belief model in primary health care centers
Source: BMC Prim Care. 2023 Dec 18;24:278. doi: 10.1186/s12875-023-02248-6 (PMC10729541; doi:10.1186/s12875-023-02248-6)
Supplement: Supplementary file 1 — Supplementary Material 1: An example of SMS sent [file 12875_2023_2248_MOESM1_ESM.pdf]

Questionnaire (translated from Persian)

Hello and thank you for giving us your time. The present questionnaire is related to the consumption of fruits and vegetables related to the prevention of cardiovascular diseases. The information obtained from this questionnaire will be kept with the researcher and there is no need to write the name and family name. Thank you in advance for your cooperation.

Demographics:

1. Gender: male☐ female☐

1. Age: .... year

2. Education level: elementary school☐ middle school☐ diploma☐ university☐

3. Size of the household: .... people

4. Marital status: single☐ married☐

5. Job: ....

6. Average monthly household income: ……...…… Toman

7. Smoking..................................

<u>Choose one option for each question</u>

Knowledge:

1. What is the most important cause of cardiovascular diseases?

A. Improper nutrition☐

B. Lack of physical activity☐

C. smoking☐

E. Several factors play a role in causing the disease☐

F. I do not know☐

2. Which sentence is correct about cardiovascular diseases?

A: A deadly disease that cannot be prevented☐

B: It is a disease that is difficult to treat, but it is preventable☐

C: It is a hereditary disease that cannot be prevented or treated☐

D: I do not know☐

3. Which sentences is correct about the prevention of cardiovascular diseases?

A: Cardiovascular diseases are not preventable☐

B: At the time of feeling suspicious symptoms, by treating it, cardiovascular diseases can be prevented☐

C: You should think about preventing cardiovascular diseases throughout your life☐

4. Fruits and vegetables mostly contain which of the following substances?

A. Fat☐ B. Vitamin☐ C. Protein☐ D. I do not know☐

5. How much fruit should be consumed daily?

A. 2-3 units☐ B. 3-5 units☐ C. 2-4 units☐ D. I do not know☐

6. How much vegetables should be consumed daily?

A. 2-3 units☐ B. 3-5 units☐ C. 2-4 units☐ D. I do not know☐

Perceived susceptibility:

1) I am worried about getting cardiovascular diseases.

A: I completely disagree☐ B: I disagree☐ C: I have no opinion☐ D: I agree☐ E: I completely agree☐

2) I am sure that I will not suffer from cardiovascular diseases

A: I completely disagree☐ B: I disagree☐ C: I have no opinion☐ D: I agree☐ E: I completely agree☐

3) If I don't consume enough fruits and vegetables, I may get cardiovascular diseases.

A: I completely disagree☐ B: I disagree☐ C: I have no opinion☐ D: I agree☐ E: I completely agree☐

4) The history of cardiovascular diseases in the family and relatives increases the probability of getting it in me.

A: I completely disagree☐ B: I disagree☐ C: I have no opinion☐ D: I agree☐

E: I completely agree☐

Perceived severity:

1) Cardiovascular diseases can lead to my death

A: I completely disagree☐ B: I disagree☐ C: I have no opinion☐ D: I agree☐

E: I completely agree☐

2) In case of suffering from cardiovascular diseases, due to the involvement of all the organs of the body, serious complications will occur in me

A: I completely disagree☐ B: I disagree☐ C: I have no opinion☐ D: I agree☐

E: I completely agree☐

3) In case of suffering from cardiovascular diseases, there will be heavy economic costs for me and my family

A: I completely disagree☐ B: I disagree☐ C: I have no opinion☐ D: I agree☐

E: I completely agree☐

Perceived benefits:

1) Consuming enough fruits and vegetables will give me confidence in maintaining my health

A: I completely disagree☐ B: I disagree☐ C: I have no opinion☐ D: I agree☐

E: I completely agree☐

2) Consuming enough fruits and vegetables makes my cardiovascular system work better

A: I completely disagree☐ B: I disagree☐ C: I have no opinion☐ D: I agree☐

E: I completely agree☐

3) Consuming enough fruits and vegetables prevents many other diseases

A: I completely disagree☐ B: I disagree☐ C: I have no opinion☐ D: I agree☐

E: I completely agree☐

Perceived barriers:

1) Because I don't know the nutritional principles (use of recommended food groups), I don't follow them.

A: I completely disagree☐ B: I disagree☐ C: I have no opinion☐ D: I agree☐

E: I completely agree☐

2) Adherence to nutritional principles (use of recommended food groups) causes me to stay away from my favorite foods.

A: I completely disagree☐ B: I disagree☐ C: I have no opinion☐ D: I agree☐

E: I completely agree☐

3) Observance of nutritional principles (use of recommended food groups) is a difficult task.

A: I completely disagree☐ B: I disagree☐ C: I have no opinion☐ D: I agree☐

E: I completely agree☐

4) Fruits and vegetables are very expensive. 5) Fruits and vegetables are not compatible with my taste.

A: I completely disagree☐ B: I disagree☐ C: I have no opinion☐ D: I agree☐

E: I completely agree☐

6) I don't like fruits and vegetables.

A: I completely disagree☐ B: I disagree☐ C: I have no opinion☐ D: I agree☐

E: I completely agree☐

7) Using fruits and vegetables means changing my diet, which is difficult for me.

A: I completely disagree☐ B: I disagree☐ C: I have no opinion☐ D: I agree☐

E: I completely agree☐

Have you consumed the following foods in the past week? How many times if used?

Food item	I did not consume at all	Once	Twice	Three times	Four times	5-6 times	Everyday
Milk							
yogurt							
cream							
jam/honey							
cheese							
bread							
baguette							
rice							
all kinds of kebabs							
pasta							
chicken							
fish							
canned fish							
lentils/beans							
sausages							
eggs							
stews							
soups							
Broth							
potatoes							
pizza							
pickles							
tomatoes							
citrus fruits							
other fruits							
puffs							
sweets							
Nuts							
ice cream							
chocolate							
tea							

coffee							
soda							
date							
tamarind							
