# Supplementary material for: Promotion of nutritional behaviors in the prevention of cardiovascular diseases: application of the health belief model in primary health care centers
Source: BMC Prim Care. 2023 Dec 18;24:278. doi: 10.1186/s12875-023-02248-6 (PMC10729541; doi:10.1186/s12875-023-02248-6)
Supplement: Supplementary file 2 — Supplementary Material 2: An example of text messages sent (translated from Persian) [file 12875_2023_2248_MOESM2_ESM.pdf]

An example of text messages sent (translated from Persian)

Dear friend

Do you know that the first cause of death in our country is cardiovascular diseases?

If we want, we can prevent cardiovascular diseases.

Reduce salt consumption. Use grilled and low-fat foods instead of fatty and fried foods. Have two servings of fruits and vegetables in the daily diet. Instead of red meat, use chicken, fish and beans.

Wait for our next messages.
